# Gastrointestinal Manifestations in a Patient With Endolimax nana Infection: A Case Report

**DOI:** 10.7759/cureus.94840

**Published:** 2025-10-18

**Authors:** Mariana Castro, Joana Brandão Silva, Raquel Freitas, Ricardo Barbosa, João Beleza Bernardes

**Affiliations:** 1 Family Medicine, Marco Family Health Unit, Local Health Unit of Tâmega and Sousa, Marco de Canaveses, PRT; 2 Family Medicine, Abel Salazar Institute of Biomedical Sciences, Porto, PRT

**Keywords:** diagnosis, diarrhea, endolimax nana, family medicine, infectious diseases, public health

## Abstract

A 54-year-old female patient with a history of depressive disorder, previously treated and not currently on regular medication, lives in a rural area and regularly consumes untreated well water. She presented with a one-month history of abdominal pain, diarrhea, and tenesmus. Laboratory tests only revealed numerous *Endolimax nana* cysts in her stool, and endoscopic evaluation did not show significant findings. The patient's complete resolution of symptoms was achieved through the combined effect of metronidazole treatment and discontinuation of untreated well water consumption.

*Endolimax nana *is a generally non-pathogenic intestinal ameba, often found in untreated or contaminated water sources. However, it can be linked to gastrointestinal issues. Avoiding contaminated water is crucial for managing and preventing such infections. In cases of chronic diarrhea in people exposed to untreated water, doctors should consider parasitic infections in their differential diagnosis and start appropriate antimicrobial treatment based on the identified pathogen.

The following case report delineates the diagnostic and therapeutic strategies employed in a patient diagnosed with *Endolimax nana* infection.

## Introduction

According to the World Health Organization, in 2019, Portugal had a mortality rate of 6.8 deaths per 100,000 inhabitants related to environmental hygiene factors, water, and sanitation [1]. In the region of Marco de Canaveses, a rural city in northern Portugal, only 54% of households were served by the public water supply network in 2022, and 51% had access to wastewater sanitation [2,3]. It is assumed that well or spring water consumption continues. Drinking contaminated water can transmit various diseases, particularly gastrointestinal infections, due to protozoa such as *Giardia* and *Entamoeba histolytica*, which frequently present with diarrhea and lead individuals to seek medical care [4]. *Endolimax nana* is generally considered a non-pathogenic microorganism and is usually not associated with gastrointestinal symptoms. However, according to the literature, in cases of high parasitic load or coinfection with other microorganisms, it may be linked to clinical manifestations. This case report describes the diagnostic and therapeutic approach used in a patient with *Endolimax nana* infection.

## Case presentation

A 54-year-old woman lived with her husband and daughter in Marco de Canaveses. She had a personal history of depressive disorder that had been previously treated with sertraline. At that time, she was not taking any regular medication. She denied having any knowledge of allergies or medication intolerances. She reported no regular alcohol consumption, was a non-smoker, and did not use any other drugs. She mentioned walking for about 30 minutes three to four times a week. There was no relevant family history. She had engaged with the recommended national cancer screenings at a population level in Portugal: her breast cancer screening, colorectal cancer screening, and cervical cancer screening were up to date. The National Vaccination Plan was up to date, with no additional vaccines required.

Current illness history

Scheduled Consultation on June 17, 2024

The patient reported experiencing soft stools for the past month, with four to five episodes per day, mainly occurring after 11 AM and not waking her up at night. Associated symptoms included fatigue, nausea, and abdominal pain, primarily in the epigastric area, described as colicky and without radiation, with no identified relieving or aggravating factors, as well as bloating. She noted a weight loss of about 4 kg since the onset of symptoms. She denied hematochezia, rectal bleeding, mucus in the stool, fever, or heartburn. She reported drinking untested well water and using public sewage. She denied having pets, recent travel, antibiotic use, or contact with sick people. Additionally, she denied receiving any treatment for these symptoms.

During the physical examination, she appeared fatigued and showed signs of mild dehydration. However, she was well-perfused, afebrile, and eupneic, with no indications of respiratory distress. Her blood pressure was 131/78 mmHg, and her heart rate was 60 beats per minute. Cardiac and pulmonary auscultation revealed no significant abnormalities. The abdominal examination showed active bowel sounds, a soft and depressible abdomen, mild tenderness in the epigastric area, and no palpable masses or organomegaly. There were no signs of peritoneal irritation, and diffuse tympany was noted throughout the abdomen. Her current weight was 69 kg, compared to 70 kg recorded in 2022.

The laboratory test, conducted on May 14, 2024 (Table 1), showed a complete blood count, glucose, thyroid-stimulating hormone (TSH), aspartate aminotransferase (AST), alanine aminotransferase (ALT), and gamma-glutamyl transferase (GGT) all within normal reference ranges.

**Table 1:** Laboratory analysis results realized on May 14, 2024 ALT: alanine aminotransferase, AST: aspartate aminotransferase, Cl: chloride, Na: sodium, K: potassium, GGT: gamma-glutamyl transferase, RDW: red cell distribution width, TSH: thyroid-stimulating hormone, FT4: free thyroxine, HDL: high-density lipoprotein, LDL: low-density lipoprotein

Test name	Result	Reference value	Units
Uric acid in serum or plasma	3.7	2.4-6.0	mg/dL
ALT	17	<34	U/L
AST	16	<31	U/L
Cl	105	98-107	mmol/L
Na	140	3.5-5.1	mmol/L
K	4.5	3.5-5.1	mmol/L
Creatinine	0.69	0.6-1.1	mg/dL
Fasting glucose	80	70-126	mg/dL
Vitamin B12	469	200-900	pg/mL
Folate	21.21	>4.0	ng/mL
GGT	13	<38	U/L
Hematocrit	36.7	37-47	%
Red blood cells (erythrocytes)	4.27	4.2-5.4	10⁶/µL
Mean corpuscular volume	85.9	78.2-97.9	fL
Hemoglobin	12.0	12.0-15.5	g/dL
RDW	13.2	11.5-14.5	%
White blood cells (leukocytes)	5.220	4.000-11.000	10⁹/L
Neutrophils	2.790	1.560-6.450	10⁹/L
Eosinophils	0.120	0.030-0.480	10⁹/L
Basophils	0.050	0.10-0.080	10⁹/L
Lymphocytes	1.970	0.950-3.070	10⁹/L
Monocytes	0.290	0.260-0.810	10⁹/L
Platelets	181	150.000-400.000	10^3^µL
TSH	3.589	0.4-4.0	mIU/L
FT4	1.18	0.8-1.8	ng/dL
HDL cholesterol	63	>50	mg/dL
Total cholesterol	220	<200	mg/dL
LDL cholesterol	145	<130	mg/dL
Triglycerides	63	<150	mg/dL

A diagnosis of chronic diarrhea was made, leading to the request for parasitological and bacteriological stool tests due to the patient's consumption of untested water and recent clinical tests that showed no abnormalities. The patient was empirically treated with a probiotic (Atyflor Hidra Mais®), a single dose of albendazole 400 mg, azithromycin 500 mg once daily for three days, and omeprazole 20 mg once daily for 15 days, starting after stool collection. Non-pharmacological measures, such as hydration and dietary management, were emphasized. A follow-up appointment was scheduled in two weeks, but was missed due to a strike.

Scheduled Consultation on July 29, 2024

The patient returned for a consultation to review her test results. Since she had not improved with the prescribed therapy, she sought medical advice in a private setting after receiving the results from the parasitological stool study. The study revealed numerous *Endolimax nana* cysts, while the bacteriological stool analysis was negative. She was treated with metronidazole 250 mg every 12 hours for 10 days, which resulted in significant improvement of her symptoms. However, a few days after finishing the antibiotic therapy, her symptoms reappeared. During the physical examination, she was well-hydrated and well-perfused, and the rest of the examination remained normal. According to the CDC®, *Endolimax nana* is considered a non-pathogenic organism, which did not seem to explain her symptoms [5]. Therefore, a decision was made to order an upper digestive endoscopy and complete colonoscopy, along with repeated laboratory tests, including a complete blood count, ferritin, sedimentation rate, and C-reactive protein. Non-pharmacological measures and the discontinuation of well water consumption were emphasized again. A follow-up appointment was scheduled.

Consultation on August 9, 2024

The patient returned for an open consultation due to increased frequency of diarrhea, which retained the previously described characteristics and was accompanied by nausea. She was still awaiting a colonoscopy and did not bring the results of the earlier requested analytical study to the appointment. She continued to drink well water, and the physical examination remained unchanged. It was decided to treat her again with metronidazole 250 mg, using a different dosing regimen of 1 tablet every eight hours for 10 days, following the protocol described by Poulsen and Stensvold (2016) [6]. The remaining symptoms were managed with oral rehydration solution, probiotics (Atyflor Hidra Mais®), and 20 mg of omeprazole. A new parasitological stool analysis was ordered. The importance of avoiding well water consumption was emphasized, and understanding and adherence to this recommendation were confirmed.

Teleconsultation on August 19, 2024

The patient was on her final day of metronidazole treatment. She reported significant improvement in her symptoms, with only mild epigastric discomfort remaining. She was still waiting for the colonoscopy. The requested laboratory study showed no changes, realized in July 2024 (Table 2). It was recommended to continue 20 mg of omeprazole once daily until the endoscopic evaluation was completed.

**Table 2:** Laboratory analysis results realized in July 2024

Test name	Result	Reference value	Units
C-reactive protein	0.09	<0.5	mg/dL
Erythrocyte sedimentation rate	13	<30	mm
Hematocrit	37.8	37-47	%
Ferritin	6	10-291	ng/mL
Red blood cells (erythrocytes)	4.39	4.2-5.4	10⁶/µL
Mean corpuscular volume	86.1	78.2-97.9	fL
Hemoglobin	12.4	12.0-15.5	g/dL
Red cell distribution width	12.5	11.5-14.5	%
White blood cells (leukocytes)	5.430	4.000-11.000	10⁹/L
Neutrophils	2.900	1.560-6.450	10⁹/L
Eosinophils	0.110	0.030-0.480	10⁹/L
Basophils	0.040	0.10-0.080	10⁹/L
Lymphocytes	1.990	0.950-3.070	10⁹/L
Monocytes	0.390	0.260-0.810	10⁹/L
Platelets	190	150.000-400.000	10^3^µL

Consultation on August 30, 2024

The patient returned for an urgent family medicine consultation due to complaints of epigastric abdominal pain and heartburn, which worsened at night and after consuming fatty foods and carbonated beverages. She denied experiencing diarrhea. She was not taking a proton pump inhibitor. On physical examination, no significant findings were noted, and she was prescribed 20 mg of omeprazole once daily, with an emphasis on non-pharmacological measures.

Consultation on October 3, 2024

**Figure 1:**
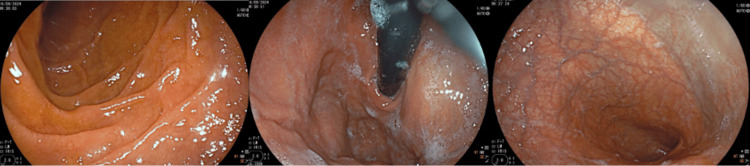
Upper endoscopic view showing the mucosal surface

**Figure 2:**
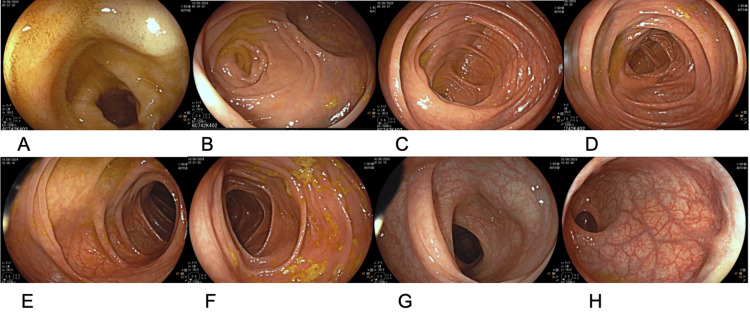
Colonoscopic images of the terminal ileum (A), cecum (B), ileocecal valve (C), ascending colon (D), hepatic flexure (E), descending colon (F), sigmoid colon (G), and rectum (H)

The patient attended the appointment to review the results of the endoscopic examination, as described in Table 3, along with the endoscopic finding images from upper endoscopy (Figure 1) and colonoscopy (Figure 2). She was asymptomatic and was taking 20 mg of omeprazole daily. The upper gastrointestinal endoscopy showed erosive gastropathy, and the biopsy was negative for *Helicobacter pylori*, with mild activity, and no atrophy, metaplasia, or dysplasia. The colonoscopy revealed no abnormalities, with adequate bowel preparation (Boston score 6). Follow-up parasitological stool analysis yielded negative results. It was decided to continue 20 mg of omeprazole once daily and to avoid well water consumption.

**Table 3:** Upper gastrointestinal endoscopy results realized on September 16, 2024

Upper gastrointestinal endoscopy			
Esophagus	Mucosa without lesions; esophagogastric junction regular and without lesions		
Stomach	Spacious and distensible; cardia adequately closed; fundus and cardiac mucosa observed in retroflexion, without lesions; body mucosa with atrophic appearance, with flattening of the mucosal folds and increased vascular pattern; antrum and incisura mucosa without lesions; pylorus centered and patent.		
Duodenum	Bulb without lesions; second portion of the duodenum without lesions		
Histopathology			
Gastric biopsy fragment (antral type mucosa)		Mild chronic gastritis with mild activity, no glandular atrophy, no intestinal metaplasia, no dysplasia, and negative for Helicobacter pylori	
Gastric biopsy fragment (body type mucosa)			Moderate chronic gastritis with moderate activity, no glandular atrophy, no intestinal metaplasia, no dysplasia, and negative for Helicobacter pylori
Conclusion: Mild to moderate chronic gastritis			

## Discussion

*Endolimax* is generally considered a non-pathogenic commensal protozoan that inhabits the human colon [5]. It has an estimated global prevalence of 13.4%, but in some continents, such as Africa, the prevalence is estimated to be around 80% [7]. Its transmission occurs via the fecal-oral route through the consumption of contaminated water or food. It is commonly found in deep well water and raw vegetables [8]. Its mechanism of action may involve irritating the intestinal mucosal crypts. It may present as acute or chronic diarrhea, abdominal pain, nausea, vomiting, flatulence, and anorexia [9,10]. Diagnosis is made through microscopic examinations [5,6,11].

Poulsen and Stensvold (2016) mention that *Endolimax* shows little evidence of pathogenicity [6]. However, the literature suggests that symptoms depend on host immunity, parasitic load, genetic variability, and possible coinfection with other fecal-contaminating microorganisms [4,6,12].

According to Poulsen and Stensvold (2016), the recommended treatment is metronidazole 250 mg three times a day for 10 days or diphetarsone 500 mg three times a day for 10 days, which is not available in community pharmacies in Portugal. In cases of known coinfection, it is advised to treat the other pathogenic agents [6,11,13].

In this case, the epidemiology, regular consumption of well water, absence of other abnormalities in diagnostic tests, and response to metronidazole 250 mg three times a day suggest that the patient's symptoms were possibly associated with an *Endolimax nana *infection. The authors believe that treatment with metronidazole 250 mg twice daily for 10 days was insufficient in this case, as symptoms improved but did not resolve. Complete resolution was achieved only after increasing the dosage to three times daily in combination with avoiding the intake of untreated well water. However, the therapeutic success cannot be attributed solely to the higher daily dose, as both measures were likely contributory.

This case demonstrates the importance of family doctors understanding the habits and sanitary conditions of the population in their practice area. Marco de Canaveses has low coverage rates for sanitation and public water supply. With this information, a more comprehensive clinical assessment was possible, leading to a quicker resolution of the case.

## Conclusions

This case highlights the importance of considering environmental factors in diagnosing and managing gastrointestinal complaints. There is limited information available in the literature. Although *Endolimax nana* is traditionally considered non-pathogenic, the patient's epidemiological context, response to treatment, and lack of other identifiable causes suggest it played a role in their symptoms. This situation underscores the need for appropriate antimicrobial treatment and improvements in sanitation as part of public health efforts. The patient's recovery, achieved through the combined effect of pharmacological treatment and the avoidance of contaminated well water, illustrates the One Health concept, which highlights the interdependence between human health, environmental conditions, and potential animal reservoirs. It emphasizes the family physician's role in evaluating both medical and environmental factors to provide better patient care. This report also emphasizes the limitations of single-case evidence and underscores the need for future case series or controlled studies to better understand the pathogenic potential of *Endolimax nana*.

## References

[1] (2024). World Health Organization: Data: Mortality rate attributed to exposure to unsafe WASH services (per 100 000 population). WASH services (per.

[2] (2025). Instituto Nacional de Estatística: Proportion of dwellings served by water supply (%) by geographic location. https://www.ine.pt/xportal/xmain?xpid=INE&xpgid=ine_unid_territorial&menuBOUI=13707095&contexto=ut&selTab=tab3.

[3] (2025). Instituto Nacional de Estatística: Proportion of dwellings served by wastewater treatment (%) by geographic location. https://www.ine.pt/xportal/xmain?xpid=INE&xpgid=ine_indicadores&indOcorrCod=0009605&xlang=pt&contexto=bd&selTab=tab2.

[4] (2025). World Health Organization: Drinking-water. https://www.who.int/news-room/fact-sheets/detail/drinking-water.

[5] (2025). Centers for Disease Control and Prevention: Intestinal (non-pathogenic) amebae. https://www.cdc.gov/dpdx/intestinalamebae/index.html.

[6] Poulsen CS, Stensvold CR (2016). Systematic review on Endolimax nana: a less well studied intestinal Ameba. Trop Parasitol.

[7] Kantzanou M, Karalexi MA, Vrioni G, Tsakris A (2021). Prevalence of intestinal parasitic infections among children in Europe over the last five years. Trop Med Infect Dis.

[8] Falcone AC, Navone GT (2023). Endolimax nana (non-pathogenic intestinal parasite). Parasitic Protozoa of Health Importance: A Transdisciplinary Approach (Book in Spanish).

[9] Hooshyar H, Arbabi M, Rostamkhani P (2023). Checklist of Endolimax species recognized in human and animals, a review on a neglected intestinal parasitic amoeba. Iraq Med J.

[10] Zeinali S, Rezgi M, Gholinejad M, Jafari R (2023). A large-scale study on the prevalence of intestinal parasites in patients referred to medical laboratories in Urmia, Northwest Iran. BMC Gastroenterol.

[11] Veraldi S, Angileri L, Rossi LC, Nazzaro G (2020). Endolimax nana and urticaria. J Infect Dev Ctries.

[12] Shah M, Tan CB, Rajan D, Ahmed S, Subramani K, Rizvon K, Mustacchia P (2012). Blastocystis hominis and Endolimax nana co-infection resulting in chronic diarrhea in an immunocompetent male. Case Rep Gastroenterol.

[13] Elliott DE (2000). Parasitic infections of the small intestine. Curr Treat Options Gastro.

